# Lack of Association of rs1192415 in *TGFBR3-CDC7* With Visual Field Progression: A Cohort Study in Chinese Open Angle Glaucoma Patients

**DOI:** 10.3389/fgene.2018.00488

**Published:** 2018-10-24

**Authors:** Yuhong Chen, Chen Qiu, Shaohong Qian, Junyi Chen, Xueli Chen, Li Wang, Xinghuai Sun

**Affiliations:** ^1^Department of Ophthalmology and Vision Science, Eye and Ear Nose Throat Hospital, Shanghai Medical College, Fudan University, Shanghai, China; ^2^Key Laboratory of Myopia, Ministry of Health, Shanghai, China; ^3^Key Laboratory of Visual Impairment and Restoration, Shanghai, China; ^4^State Key Laboratory of Medical Neurobiology, Institute of Brain Science, Fudan University, Shanghai, China

**Keywords:** POAG, visual field, progression, *TGFBR3-CDC7*, *TMCO1*, *ATOH7*, *CDKN2B-AS1*, *SIX1/SIX6*

## Abstract

To investigate the association of known candidate genes with the visual field (VF) progression of primary open angle glaucoma (POAG) in a Han Chinese population. We included 440 POAG patients in this study. Fourteen previously reported single nucleotide polymorphisms (SNPs) at five different gene regions (*TGFBR3-CDC7*, *TMCO1*, *CDKN2B-AS1*, *ATOH7*, and *SIX1/SIX6*) were genotyped. Age at diagnosis, gender, intraocular pressure (IOP), mean defect (MD) of VF, vertical cup disk ratio (VCDR), best corrected visual acuity (BCVA), central corneal thickness (CCT), and axial length (AL) were recorded at baseline. Patients were followed up for 5 years to evaluate VF progression over time. Clinical information and allele frequencies of 14 SNPs were compared between patients who progressed and who did not within 5 years by multivariate logistic regression. Survival analysis was performed to evaluate the contribution of the associated SNP by cox regression. Greater MD (*P* < 0.0001), increased VCDR (*P* = 0.0001), higher IOP (*P* = 0.0003), worse BCVA (*P* = 0.002), and older age (*P* = 0.030) at the baseline were associated with VF progression. Both multivariate logistic regression and cox regression survival analysis showed none of the 14 SNPs statistically associated with VF progression adjusted with age at diagnosis, gender, baseline MD, follow-up IOP, CCT, and AL. There were lack of association of SNPs at *TGFBR3-CDC7*, *TMCO1*, *ATOH7*, *CDKN2B-AS1*, *SIX1/SIX6* loci with VF progression in POAG patients in Han Chinese. Further studies are needed to evaluate the association of genetic variants with VF progression.

## Introduction

Glaucoma is the leading cause of irreversible blindness all over the world and primary open angle glaucoma (POAG) is the most common type of glaucoma. The estimate for blindness of POAG at 15 years was 14.6% in one eye and was 6.4% in both eyes from a relatively recent study (Chen, 2003). There is enormous variability in progression and outcome among POAG patients. To clarify the factors associated with disease progression is very important in improving the prognosis of POAG. Although previous studies have revealed older age, higher intraocular pressure (IOP), greater visual field (VF) loss, the presence of optic disk hemorrhage and other risk factors are associated with VF progression (Leske et al., 2003, 2007; Musch et al., 2009; Prata et al., 2010; De Moraes et al., 2011; Kim et al., 2015), identifying molecular genetic factors underlying POAG progression in further would be helpful to understand the pathogenic mechanism of the disease at a molecular level and potentially develop method to control the disease.

Many genes were identified to be significantly associated with POAG, such as *CDKN2B-AS1* (cyclin-dependent kinase inhibitor 2B antisense RNA 1) (Burdon et al., 2011, 2012), *TMCO1* (transmembrane and coiled-coil domain 1) (Burdon et al., 2011; Wiggs et al., 2012), *SIX1-SIX6* (sin oculis homeobox 1/sin oculis homeobox 6) (Fan et al., 2011; Wiggs et al., 2012), *ATOH7* (atonal bHLH transcription factor 7) (Ramdas et al., 2011), *TGFBR3-CDC7* (transforming growth factor beta receptor 3- cell division cycle 7) (Khor et al., 2011; Li et al., 2015), and et al.

Interestingly, these genes are functionally involved in pathways of optic nerve development and retinal ganglion cell (RGC) apoptosis (Wiggs, 2015) as well. *ATOH7* and *SIX1/SIX6* play important roles in ocular development. *CDKN2B* and *TMCO1* are involved in cell cycle maintenance and apoptosis of RGCs. Both *TGFBR3* and *CDC7* are involved in transforming growth factor b (TGF-β) signaling pathway (Shi et al., 2012). TGF-β2 may be important in the development of glaucoma, since it has been implicated high levels in glaucomatous optic nerve damage and RGC death (Pena et al., 1999), as well as in the aqueous humor. *CDC7* encodes a cell division cycle protein which is the TGF-β downstream targets mediating both the differentiation and proliferation.

Therefore, both genetic data and functional data imply their contribution to the progressive degeneration of optic nerve in POAG. However, whether they are associated with the progression of glaucoma is still unclear. The studies about the association of genetic variants with VF progression are rare, partly because such longitudinal VF progression data with genetic data is not easy to obtain. Trikha et al. (2015) recently found the presence of the index single nucleotide polymorphism (SNPs) rs1192415 (*TGRBR3-CDC7*) was associated with VF progression in POAG patients from Singapore. It would be interesting to validate this SNP in an independent population. Furthermore, exploration of other associated SNPs at the other candidate gene loci is also important for clarification of the genetic contribution of candidate genes for VF progression further.

In this study, we investigated whether published genetic markers of POAG associated with VF progression by following a Han Chinese cohort, in order to evaluate the contribution of genetic factors to the progression of glaucoma.

## Materials and Methods

This study was approved by the Ethics Review Committee of Eye and Ear, Nose, Throat Hospital (EENT), Fudan University. The design and implement of this study adhered to the tenets of the Declaration of Helsinki. Written informed consent was obtained from all the patients.

### Study Cohort

Patients were enrolled in the Eye and ENT hospital, Fudan University from 2007 to 2012 and were followed up for 5 years. POAG was defined as glaucomatous optic neuropathy compatible with VF loss in at least one eye and coupled with open angles on gonioscopy for both eyes. The glaucomatous optic neuropathy was defined as a vertical cup disk ratio (VCDR) > 0.7 or an inter-eye asymmetry of > 0.2, with notching, rim thinning, or retina nerve fiber layer (RNFL) defect. A minimal glaucomatous VF defect was defined as presence of at least three contiguous non-edged test points within the same hemifield on the corrected probability plot at *P* < 0.05, with at least one point *P* < 0.01, excluding points directly above and below the blind spot. Patients with initial IOP > 21 mmHg were diagnosed as high tension glaucoma (HTG), while the patients with IOP ≤ 21 mmHg were diagnosed as normal tension glaucoma (NTG).

Secondary glaucoma, such as pigmentary, uveitic, neovascular, traumatic glaucoma and glucocorticoid induced glaucoma, were excluded. Patients with concomitant ocular diseases, which could potentially impair VF, such as optic disk anomalies, optic nerve diseases, retinal diseases, pathologic myopia, and intracranial lesions were excluded as well. Other exclusion criteria included history of intraocular surgery or refractive laser therapy, best corrected visual acuity (BCVA) less than 20/40, and mean defect (MD) of VF worse than 20 dB.

### Clinical Examination

Each Patient underwent a complete eye examination at the baseline including BCVA, slit lamp examination of the anterior chamber, gonioscopy and fundus, measurement of IOP, central corneal thickness (CCT), axial length (AL), VCDR, and VF. Goldmann applanation tonometry was used to measure IOP. Three independent IOPs were examined at different time before treatment for all the patients. Average untreated IOP at presentation was considered as the baseline IOP. Follow-up IOP was the average of IOPs at all follow-up visits until progression or end of follow-up. CCT and AL were measured by low-coherence interferometry (LenStar 900; Haag-Streit, Köniz, Switzerland). Stereoscopic photographs of the optic disk were taken by fundus camera (EOS D60 digital camera, Canon, Utsunomiyashi, Tochigi Prefecture, Japan). VFs were examined by Octopus (G2 program, Octopus 101, Haag-Streit, Inc., Köniz, Switzerland). The optic disk photos and VFs were read by two experienced ophthalmologists (X.S. and S.Q.) independently and a third ophthalmologist (J.C.) would be consulted if disagreement existed. Demographic features, such as age, gender and family history were also recorded.

Patients were followed up every month for the first 3 months after diagnosis and after that were examined VF for every 6 months. All the patients received a conventional course of medical therapy, in which a monotherapy was initially used and then prescribed combination of topical hypotensive agents if target IOPs were not reached. When the glaucoma could not be controlled even with the maximum tolerated medical treatment, glaucoma surgery or laser treatment was recommended, which came to the endpoint of our follow up. Patients were followed up for at least 5 years.

### Visual Field Analysis

Visual field Progression was analyzed using reliable VF tests (both false positive and negative catch trials under 15% and the reliability factor under 15). VF progression was defined according to the event-based analysis modified for Octopus perimetry (Hodapp et al., 1993; Naghizadeh and Hollo, 2014). Briefly, VF was considered deteriorated when satisfying at least one of the following criteria: (a) developing a new scotoma of at least three non-edge points worsening ≥ 5 dB, or one non-edge point worsening ≥ 10 dB; (b) a cluster of ≥ 3 non-edge points with ≥ 10 dB deteriorating in a preexisting scotoma; (c) developing a new cluster of ≥ 3 non-edge points with 15 degree around a preexisting scotoma; (d) worsening of the global MD value by ≥ 2 dB/y. The first progressing eye of each patient was included for analysis. If both eye progressed at the same time, the greater progressed eye was included. If neither eye progressed, the eye with worse VF was included.

### Genotyping

Genomic DNA was extracted from leukocytes of the peripheral blood for each participant. It was purified by the Qiagen QIAmp Blood Kit (Qiagen, Hilden, Germany). Fourteen previously reported SNPs were chosen for genotyping, including rs1900004, rs3858145, rs7916697 at *ATOH7*, rs10116277, rs1063192, rs2157719, rs4977756, rs523096, rs7049105 at *CDKN2B-AS1*, rs33912345, and rs10483727 at *SIX1/SIX6*, rs4656461, and rs7555523 at *TMCO1*, as well as rs1192415 at *TGFEB3-CDC7*. The SNPs included in this study were either tagging SNPs referenced to HapMap database or representative SNPs reported significantly associated with development of POAG in previous publications (Burdon et al., 2011, 2012; Fan et al., 2011; Khor et al., 2011; Ramdas et al., 2011; Wiggs et al., 2012; Li et al., 2015).

Single nucleotide polymorphism genotyping was performed using iPLEX Gold chemistry on the MassARRAY system (Sequenom, Inc., San Diego, CA, United States) by means of matrix assisted laser desorption ionization time-of-flight mass spectrometry method (MALDI-TOF) according to the manufacturer’s instructions. Genotype calling was performed in real time with MassARRAY RT software version 3.0.0.4 and analyzed using the MassARRAY Typer software version 3.4 (Sequenom). Each SNP with call rate greater than 95% was analyzed in the next step.

### Statistical Analysis

Statistical analysis was performed using STATA (version 8.0, Stata Corporation, College Station, TX, United States). Genotype and allele frequencies were calculated for each SNP. All genotyping results were screened for deviations from Hardy–Weinberg equilibrium (HWE) (*P* > 0.01). Continuous variables were expressed as mean ± SD if in accord with normal distribution and compared between progress group and non-progress group by using a Student’s *t*-test; continuous variables with abnormal distribution were expressed as median (interquartile range 25–75%) and compared between two groups using Wilcoxon Rank-Sum test. Categorical variables were compared between groups by using fisher exact test.

Multivariate logistic regression was used to calculate odds ratios (OR) with 95% confidence intervals (CI) adjusted for possible associated covariates, including age at diagnosis, gender, baseline MD, follow-up IOP, CCT, and AL. Since the VCDR and BCVA are highly related with the baseline MD but less informative and less accurate as baseline MD. Similarly, the follow-up IOP and baseline IOP were highly related to each other, and follow-up IOP was a more stable parameter than the baseline IOP. Thus, VCDR, BCVA and baseline IOP were not included in the multivariate analysis. Individual SNP genotypes were coded according to the number of copies of the risk alleles: 0 for the wild-type genotype, 1 for heterozygous carriers of the risk allele, and 2 for homozygous for the risk alleles.

Cox regression was used to analyze the contribution of the risk alleles of SNPs on the progression rate at the 5^th^ year adjusted with age at diagnosis, gender, baseline MD, follow-up IOP, CCT, and AL. A probability value of < 0.05 was defined for the statistical significance.

Linkage disequilibrium (LD) patterns and haplotype blocks were deduced using Haploview (version 4.2). Sample size and power were calculated by using a statistical tool QUANTO (version 1.2)^1^ under the assumptions of additive genetic model and parameters referenced to the previous studies (Li et al., 2015; Trikha et al., 2015). A power of 80% was achieved to detect significant difference from progressors and non-progressors with current sample size of our study.

## Results

A total of 440 POAG patients, 161 males and 279 females, with mean age of 45 (32–58) years old were enrolled in this study. The mean CCT was 540.82 ± 34.86 μm and the average AL was 25.18 ± 1.61 mm. The mean baseline IOP was 22.5 (20–28) mmHg. Two hundred and seventy six patients were diagnosed as HTG and 164 patients were diagnosed as NTG according to the baseline IOP. Patients were followed up for 5.67 (5–7) years with a median of 13 (11–15) times of VF examination.

The median baseline MD was 6.5 (3.5–10.0) dB. Based on the VF progression criteria, 191 patients fulfilled VF progression within 5 years’ follow-up while 249 patients didn’t. There was no statistically significant difference of sex ratio (*P* = 0.27), CCT (*P* = 0.32), AL (*P* = 0.75), and numbers of VF (*P* = 0.06) between progress group and non-progress group.

However, patients in the progress group had older age [50 (36–60) years old] than those in the non-progress group [43 (32–57) years old] (*P* = 0.03). The baseline MD of progress group [8.0 (5.0–11.0) dB] was greater than that of non-progress group [5.0 (3.0–8.7) dB] (*P* < 0.0001), the VCDR of progress group [0.8 (0.75–0.9)] was larger than that of non-progress group [0.8 (0.7–0.85] (*P* = 0.0001), and the BCVA of progress group [0.22 (0–0.51)] was worse than that of non-progress group [0 (0–0.22)] (*P* = 0.002). The differences of the three parameters’ (baseline MD, VCDR, BCVA) indicated patients with progression were at relatively more advanced stages at the baseline compared to patients without progression. Furthermore, progress group was observed with both higher baseline IOP [24.0 (20.4–28.8) mmHg] and follow-up IOP (16.71 ± 2.61 mmHg) than non-progress group [22.0 (19.0–26.0) mmHg and 15.73 ± 2.38 mmHg respectively, *P* = 0.0003 and 0.0001 respectively], and progress group had more proportion of HTG patients than non-progress group had (69.63 vs. 57.43%, *P* = 0.01). The clinical and demographical features of the cohort and two groups were listed in Table 1.

**Table 1:** Demographical features and basic clinical information of all, progressed and non-progressed POAG patients.

Demographical features and clinical information	Total (*n* = 440)	Progress (*n* = 191)	Non-progress (*n* = 249)	*P*-values
Age at diagnosis (years)	45 (32–58)	50 (36–60)	43 (32–57)	**0.030**
Male:Female	279:161	127:64	152:97	0.272
HTG:NTG	276:164	133:58	143:106	**0.010**
CCT (μm)	540.82 ± 34.86	538.93 ± 34.84	542.27 ± 34.89	0.323
AL (mm)	25.18 ± 1.61	25.16 ± 1.68	25.21 ± 1.55	0.746
BCVA (LogMAR)	0.22 (0–0.36)	0.22 (0–0.51)	0 (0–0.22)	**0.002**
VCDR	0.8 (0.7–0.9)	0.8 (0.75–0.9)	0.8 (0.7–0.85)	**0.0001**
Baseline IOP (mmHg)	22.5 (20.0–28.0)	24.0 (20.4–28.8)	22.0 (19.0–26.0)	**0.0003**
Follow-up IOP (mmHg)	16.15 ± 2.53	16.71 ± 2.61	15.73 ± 2.38	**0.0001**
Baseline MD (dB)	6.5 (3.5–10.0)	8.0 (5.0–11.0)	5.0 (3.0–8.7)	**<0.0001**
Follow-up time (years)	5.7 (5.0–7.0)	5.5 (5.0–6.5)	6.0 (5.0–7.5)	**0.007**
Numbers of VF	13 (11–15)	13 (10–15)	13 (11–15)	0.059

*Continuous variables in accord with normal distribution were expressed as mean ± SD and those with abnormal distribution were expressed as median (interquartile range 25–75%). The P-values represent differences between progressors and non-progressors, P-values less than 0.05 are shown in bold.*

As expected, the follow-up time of progress group [5.5 (5.0–6.5) years] was a little bit shorter than non-progress group [6.0 (5.0–7.5) years] (*P* = 0.007), as a proportion of patients in progress group quitted the study because of the invasive treatment due to uncontrolled IOPs.

All SNPs passed quality control and genotyping efficiency criteria (>95% with all the samples) and were in HWE (*P* > 0.01). ORs and *P*-values for the association between each SNP and VF progression listed in Table 2 were adjusted by age, gender, baseline MD, follow-up IOP, CCT, and AL. However, none of the 14 SNPs passed statistical significance level with VF progression.

**Table 2:** The association of single nucleotide polymorphisms and visual field progression.

Gene	SNP	Chr	Position	MA	MAF	HWE	OR (95%CI)	*P*_logit^†^	*P*_cox^‡^
*TGFBR3-CDC7*	rs1192415	1	91611540	G	0.165	0.165	1.37 (0.93–2.01)	0.108	0.277
*TMCO1*	rs4656461	1	165717968	G	0.018	0.130	2.25 (0.83–6.08)	0.112	0.326
*TMCO1*	rs7555523	1	165749742	C	0.018	0.130	2.24 (0.82–6.06)	0.114	0.338
*CDKN2B-AS1*	rs1063192	9	22003368	G	0.163	0.862	1.18 (0.80–1.74)	0.399	0.216
*CDKN2B-AS1*	rs523096	9	22019130	G	0.088	0.128	1.35 (0.83–2.21)	0.223	0.190
*CDKN2B-AS1*	rs7049105	9	22028802	A	0.319	0.511	1.18 (0.87–1.61)	0.277	0.249
*CDKN2B-AS1*	rs2157719	9	22033367	G	0.084	0.217	1.44 (0.87–2.36)	0.156	0.172
*CDKN2B-AS1*	rs4977756	9	22068653	G	0.194	1.000	1.33 (0.93–1.91)	0.119	0.070
*CDKN2B-AS1*	rs10116277	9	22081398	G	0.270	0.545	1.17 (0.85–1.62)	0.344	0.774
*ATOH7*	rs7916697	10	68232096	A	0.377	0.479	1.28 (0.96–1.71)	0.099	0.126
*ATOH7*	rs1900004	10	68241124	A	0.377	0.614	1.24 (0.93–1.67)	0.147	0.190
*ATOH7*	rs3858145	10	68252081	G	0.376	0.919	1.28 (0.95–1.72)	0.107	0.067
*SIX1/SIX6*	rs33912345	14	60509819	A	0.180	0.520	1.08 (0.75–1.56)	0.673	0.579
*SIX1/SIX6*	rs10483727	14	60606157	C	0.184	0.525	1.12 (0.78–1.61)	0.533	0.567

*Chr, chromosome; MA, minor allele; MAF, minor allele frequency; HWE, Hardy–Weinberg equilibrium; OR, odds ratio; CI, confidence interval. The position of each SNP was referred to NCBI build GRCh38.p7. ^†^P_logit values were calculated by logistic regression adjusted with age, gender, baseline MD, follow-up IOP, CCT, AL from comparison of allele frequencies between progress group and non-progress group.^‡^P_cox values were calculated by cox regression adjusted with age, gender, baseline MD, follow-up IOP, CCT, AL from comparison of patients with 1 or 2 risk alleles versus with 0 risk allele.*

Furthermore, multivariate cox proportional hazard analysis still showed no statistically significant difference of survival time with 1 or 2 risk alleles versus 0 risk allele in all the SNPs adjusted with following covariates including age, gender, baseline MD, follow-up IOP, CCT, and AL.

Linkage disequilibrium blocks were estimated for *ATOH7* and *CDKN2B-AS1* based on genotyped SNPs in these two genes (Supplementary Figures S1, S2). No association of any LD block with VF progression was detected by haplotype association tests, which were consistent with the results of single SNP association (Supplementary Table S1).

## Discussion

Glaucoma is an irreversible eye disease that naturally leads to severe visual function loss if untreated. However, the progression could varied hugely from patient to patient. In our study, both baseline IOP and follow-up IOP were strongly associated with glaucoma progression, which is consistent with previous studies showing that the IOP has the greatest impact on glaucoma progression (The Advanced Glaucoma Intervention Study [AGIS], 2000; Leske et al., 2003). However, a portion of patients continue to lose visual function even though the IOP is well-controlled, clarifying risk factors other than IOP is important to control the VF progression. Our results validated the association of older age, increased VCDR, greater baseline MD, poorer baseline BCVA with glaucoma VF progression. MD, VCDR, and BCVA were parameters mainly related with the severity of the POAG, which basically showed the worse status the patient started with, the faster progress the patient would have.

Additionally, our results showed there were significantly (*P* = 0.01) larger proportion of patients diagnosed with NTG than HTG in non-progress group (106/249, 42.6%) compared to progress group (58/191, 30.4%) and the prevalence of progression at the 5^th^ year for NTG (35.4%) was lower than that of HTG (48.2%). The results were unsurprisingly supporting the general viewpoint that most NTG patients tend to progress more slowly than HTG patients (Anderson et al., 2001; Heijl et al., 2009).

In this study, we tried to evaluate the potential contribution of five different genes (*TGFBR3-CDC7*, *TMCO1*, *CDKN2B-AS1*, *ATOH7*, and *SIX1/SIX6*) to VF progression of POAG patient with 14 known associated index SNPs. Unfortunately, none of the 14 SNPs showed statistically significant association with VF progression. Our results were unable to confirm the association of index SNP rs1192415 at *TGFBR3-CDC7* loci with VF progression, as reported in the study of Trikha et al. (2015), which showed among the loci of *CDKN2B-AS1, SIX1-SIX6, CAV1-CAV2, ABCA1, GAS7, AFAP1, GMDS*, *PMM2*, only *TGFBR3-CDC7* (index SNP rs1192415) was associated with VF progression in POAG patients from Singapore.

The discordance could be firstly due to the different patients’ population. The contribution of the SNP in *TGFBR3-CDC7* to glaucoma varies in different populations. The association *P*-value of rs1192415 was 0.01 in Europeans, while it reached 1.48 × 10^−7^ in Asian with similar sample size (Li et al., 2015). Even in different part of China, the odds ratio (OR) of this SNP varies widely (Li et al., 2015). Secondly, the relative mild association of rs1192415 with POAG and its potential mild contribution to POAG progression could also increase the difficulty of detecting a positive result. The effect size of the risk allele G was relatively small with the OR of 1.13 and *P*-value of 1.6 × 10^−8^ even from the meta analysis of 12677 POAG cases versus 36526 controls (Li et al., 2015). Thus, our limited sample size could be underpowered if the contribution of the SNP to the progression of glaucoma turned out to be very weak. Further possible reason could be due to the different design. Trikha’s study was retrospective in nature and although the initial study sample was large, many patients were excluded for less than five VF tests. The relatively fewer average times of VF examination and lower VF progression (14.5%) in 5 years than usual (Ahrlich et al., 2010; Araie et al., 2012) indicated there might be bias for that population (Trikha et al., 2015).

The *TGFBR3-CDC7* intergenic region was firstly identified to be associated with optic disk area (Khor et al., 2011) and later was verified significant association with POAG (Li et al., 2015) in large sample size of population study. SNPs at *SIX1/SIX6* loci were first reported to be strongly associated with an increased VCDR (Ramdas et al., 2010), and then were shown to be associated with POAG (Ramdas et al., 2011; Wiggs et al., 2012; Iglesias et al., 2014) as well. Although *ATOH7* was only shown suggestively associated with POAG, it has been reported to be strongly associated with optic disk area and VCDR as well (Macgregor et al., 2010). Both *TMCO1* and *CDKN2B-AS1* contributed to severe forms of glaucoma (Burdon et al., 2011). Subsequent studies suggested that the *CDKN2B-AS1* region was the most significant associated gene with POAG across different ethnic populations, especially in NTG patients (Mabuchi et al., 2012; Nakano et al., 2012; Wiggs et al., 2012; Chen et al., 2015). Instead, TMCO1 seems to be related to increased IOP (van Koolwijk et al., 2012; Chen et al., 2015). Moreover, these genes were also demonstrated to play important roles in optic nerve development and RGC apoptosis (Wiggs, 2015; Supplementary Table S2).

Therefore, all the gene loci (*TGFBR3-CDC7, TMCO1, CDKN2B-AS1, ATOH7*, and *SIX1/SIX6*) included in our study were either associated with POAG or the subphenotype of POAG, such as VCDR and IOP, and were likely to contribute to the development of POAG by given genetic evidences and functional data. It is reasonable to propose that they may contribute to the progression of POAG as well, since the same risk alleles for POAG development are possibly implicated in the progression of the same disease. However, current study did not support the association of risk alleles in associated genes with glaucoma progression. It suggested the underlying mechanisms of progression might be different from the mechanisms of development. Risk factors that influence the rate of POAG progression may not coincide with the risk factors for having POAG in the first place, which emerge from analyses of prevalence data rather than progression data. The pathogenic mechanism for progression of POAG may be multifactorial and complex with the interference of treatment.

## Conclusion

There were lack of association of *TGFBR3-CDC7, TMCO1, CDKN2B-AS1, ATOH7*, and *SIX1/SIX6* with VF progression in our POAG patients. Further studies evolving other SNPs or genes and larger sample size are needed to clarify the contribution of genetic factors to the VF progression of POAG.

## Author Contributions

YC, CQ, and XS conceived and designed the projects. XS, SQ, JC, XC, and LW provided patients’ data. YC analyzed SNPs. YC and CQ wrote the paper and patients’ follow-ups. YC, CQ, XS, and SQ reviewed and edited the paper.

## Conflict of Interest Statement

The authors declare that the research was conducted in the absence of any commercial or financial relationships that could be construed as a potential conflict of interest.

## Footnotes

1 http://biostats.usc.edu/Quanto.html

## Supplementary Material

FIGURE S1 The LD plot of 7 SNPs in *ATOH7*. Darker shades of red indicate higher values of the LD coefficient (D′). The numbers listed in each square represent the D′ value for pairwise analysis.

FIGURE S2 The LD plot of 6 SNPs in *CDKN2B-AS1.* Darker shades of red indicate higher values of the LD coefficient (D′). The numbers listed in each square represent the D′ value for pairwise analysis.

TABLE S1 The frequencies of LD blocks in *ATHOH7* and *CDKN2B-AS1* and haplotype association between progressors and non-progressors.

TABLE S2 The characteristics and the functionality of the investigated SNPs and genes.
