# Supplementary figures and images for: Lack of Association of rs1192415 in TGFBR3-CDC7 With Visual Field Progression: A Cohort Study in Chinese Open Angle Glaucoma Patients
Source: Front Genet. 2018 Oct 24;9:488. doi: 10.3389/fgene.2018.00488 (PMC6208000; doi:10.3389/fgene.2018.00488)

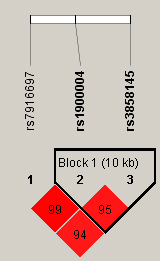

Supplement: FIGURE S1 — The LD plot of 7 SNPs in ATOH7. Darker shades of red indicate higher values of the LD coefficient (D′). The numbers listed in each square represent the D′ value for pairwise analysis. [file Image_1.JPEG]

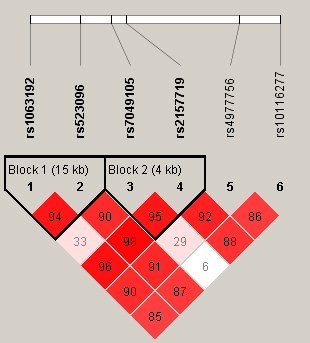

Supplement: FIGURE S2 — The LD plot of 6 SNPs in CDKN2B-AS1. Darker shades of red indicate higher values of the LD coefficient (D′). The numbers listed in each square represent the D′ value for pairwise analysis. [file Image_2.JPEG]
