# Supplementary material for: Lack of Association of rs1192415 in TGFBR3-CDC7 With Visual Field Progression: A Cohort Study in Chinese Open Angle Glaucoma Patients
Source: Front Genet. 2018 Oct 24;9:488. doi: 10.3389/fgene.2018.00488 (PMC6208000; doi:10.3389/fgene.2018.00488)
Supplement: TABLE S1 — The frequencies of LD blocks in ATHOH7 and CDKN2B-AS1 and haplotype association between progressors and non-progressors. [file Table_1.DOC]

**Supplementary Table 1.** The frequencies of LD blocks in *ATHOH7* and *CDKN2B-AS1* and haplotype association between progressors and non-progressors.

| Gene | Blocks(SNPs) | Haplotypes | Frequencies | Frequencies in progressors | Frequencies in Non-progressors | *P* Value |
| --- | --- | --- | --- | --- | --- | --- |
| *ATOH7* | Block1 (rs1900004-rs3858145) | -G-A- | 0.611 | 0.578 | 0.636 | 0.080 |
|  |  | -A-G- | 0.365 | 0.387 | 0.347 | 0.221 |
|  |  | -A-A- | 0.013 | 0.018 | 0.008 | 0.176 |
|  |  | -G-G- | 0.012 | 0.016 | 0.008 | 0.290 |
| *CDKN2B-AS1* | Block1 (rs1063192-rs523096) | -A-A- | 0.832 | 0.824 | 0.839 | 0.542 |
|  |  | -G-G- | 0.084 | 0.091 | 0.078 | 0.486 |
|  |  | -G-A- | 0.080 | 0.077 | 0.083 | 0.760 |
|  | Block2 (rs7049105-rs2157719) | -G-A- | 0.678 | 0.662 | 0.690 | 0.368 |
|  |  | -A-A- | 0.238 | 0.242 | 0.235 | 0.834 |
|  |  | -A-G- | 0.081 | 0.091 | 0.074 | 0.356 |

LD, linkage disequilibrium

The *P* values represent differences of haplotype frequencies between progressors and non-progressors
