# Supplementary material for: Lack of Association of rs1192415 in TGFBR3-CDC7 With Visual Field Progression: A Cohort Study in Chinese Open Angle Glaucoma Patients
Source: Front Genet. 2018 Oct 24;9:488. doi: 10.3389/fgene.2018.00488 (PMC6208000; doi:10.3389/fgene.2018.00488)
Supplement: TABLE S2 — The characteristics and the functionality of the investigated SNPs and genes. [file Table_2.DOC]

| Gene Symbol | Gene Name | Gene Function | SNP | Location | Exonic Function | AA change | SIFT | Polyphen2 |
| --- | --- | --- | --- | --- | --- | --- | --- | --- |
| *TGFBR3/CDC7* | transforming growth factor beta receptor 3 / cell division cycle 7 | Encoding membrane receptor involved in TGFB signaling / Encoding a cell division cycle protein with kinase activity | rs1192415 | intergenic | - | - | - | - |
| *TMCO1* | transmembrane and coiled-coil domains 1 | Encoding transmembrane protein involved in mitochondrial function and cell cycle | rs4656461 | intergenic | - | - | - | - |
|  |  |  | rs7555523 | intronic | - | - | - | - |
| *ATOH7* | atonal bHLH transcription factor 7 | A member of the basic helix-loop-helix family of transcription factors, involved in the differentiation of retinal cells and optic nerve formation | rs7916697 | 5'-UTR | - | - | - | - |
|  |  |  | rs1900004 | intergenic | - | - | - | - |
|  |  |  | rs3858145 | intergenic | - | - | - | - |
| *CDKN2B-AS1* | CDKN2B antisense RNA 1 | interacting with polycomb repressive complex-1 (PRC1) and -2 (PRC2) leading to epigenetic silencing of other genes, regulate CDKN2B and CDKN2A, involved in cell cycle | rs1063192 | ncRNA_intronic | - | - | - | - |
|  |  |  | rs523096 | ncRNA_intronic | - | - | - | - |
|  |  |  | rs7049105 | ncRNA_intronic | - | - | - | - |
|  |  |  | rs2157719 | ncRNA_intronic | - | - | - | - |
|  |  |  | rs4977756 | ncRNA_intronic | - | - | - | - |
|  |  |  | rs10116277 | ncRNA_intronic | - | - | - | - |
| *SIX1/SIX6* | SIX homeobox 1 / SIX homeobox 6 | Regulation of cell proliferation, apoptosis and embryonic development, SIX6 play a critical role in ocular development | rs33912345 | exonic | nonsynonymous | p.H141N | Tolerated | Benign |
|  |  |  | rs10483727 | intergenic | - | - | - | - |

**Supplementary Table 2.** The characteristics and the functionality of the investigated SNPs and genes.
